# Survival and Prognostic Factors for Outcome after Radiotherapy for T2 Glottic Carcinoma

**DOI:** 10.3390/cancers11091319

**Published:** 2019-09-06

**Authors:** Martine Hendriksma, Marc A.P. van Ruler, Berit M. Verbist, Martin A. de Jong, Ton P.M. Langeveld, Peter Paul G. van Benthem, Elisabeth V. Sjögren

**Affiliations:** 1 Department of Otorhinolaryngology, Head & Neck Surgery, Leiden University Medical Center, 2300 RC Leiden, The Netherlands; 2 Department of Radiation Oncology, Haaglanden Medical Center, 2300 RC The Hague, The Netherlands; 3 Department of Radiology, Leiden University Medical Center, 2300 RC Leiden, The Netherlands; 4 Department of Radiation Oncology, Leiden University Medical Center, 2300 RC Leiden, The Netherlands

**Keywords:** early glottic cancer, radiotherapy, local control, survival, anterior commissure

## Abstract

*Background:* Local recurrence after radiotherapy for T2 glottic carcinoma remains an issue and identifying patients at risk for relapse is, therefore, important. This study aimed to assess the oncological outcomes and prognostic factors in a consecutive series of patients treated with radiotherapy for T2N0 glottic carcinoma. *Methods:* Patients with T2N0 glottic carcinoma treated with radiotherapy were included in this retrospective study. Five- and ten-year local control (LC), overall survival (OS), disease-specific survival (DSS), and laryngeal preservation (LP) rates were calculated with the Kaplan–Meier method. The impact of prognostic variables was evaluated with the log-rank test. *Results:* Ninety-four patients were included for analysis. LC, OS, DSS, and LP rates were 70.5, 63.7, 86.0, and 74.7%, respectively at five years and 65.8, 41.0, 75.6, and 72.4% at 10 years. In total, 46 scans were included in the analyses. Vertical involvement of the anterior commissure on imaging showed a significant impact on LC. *Conclusions:* In accordance with previously described surgical risk factors, we identified vertical involvement of the anterior commissure on imaging as a prognostic factor for radiation failure.

## 1. Introduction

Laryngeal carcinoma is one of the most frequent types of head and neck cancer. In 2017, 708 patients were diagnosed with laryngeal carcinoma in the Netherlands. Around sixty-five percent of tumors arise from the glottic region [1], and of those, about 30% are diagnosed as T2-stage tumors [2]. The two main treatment modalities for T2 tumors are radiotherapy and transoral CO_2_ laser microsurgery (TLM). Both treatments aim to achieve high cure rates and preserve organ function with an acceptable voice outcome. The Dutch guidelines on the treatment of laryngeal carcinoma advocates radiotherapy as the treatment of choice for T2 glottic carcinoma [3]. 

Currently, there is no definite proof that one treatment modality is more effective than the other [4]. Several studies have shown that both radiotherapy and TLM provide high, comparable local control (LC) rates for T2 glottic carcinoma [5]. However, a recent systematic review on T2 glottic carcinoma showed that, similar to findings for T1a glottic carcinoma [6,7,8], larynx preservation (LP) rates after primary treatment were higher with TLM than with radiotherapy [9]. Hence, determining prognostic factors could improve risk stratification in patients considered for radiotherapy in T2 glottic carcinoma. This information could help enhance outcomes by facilitating the identification of patients most likely to benefit from radiotherapy as opposed to surgical treatment. 

The present retrospective study aimed to (1) evaluate survival outcomes in consecutive patients that received primary radiotherapy for T2N0 glottic carcinoma at our center and to compare these results to those in the current literature, as well as to (2) identify factors predictive for survival outcomes in this cohort. The ultimate goal of the study is to contribute to the ongoing evaluation of the comparative benefits of radiotherapy and TLM for patients with T2 glottic carcinoma.

## 2. Methods

### 2.1. Patients

**Figure 1:**
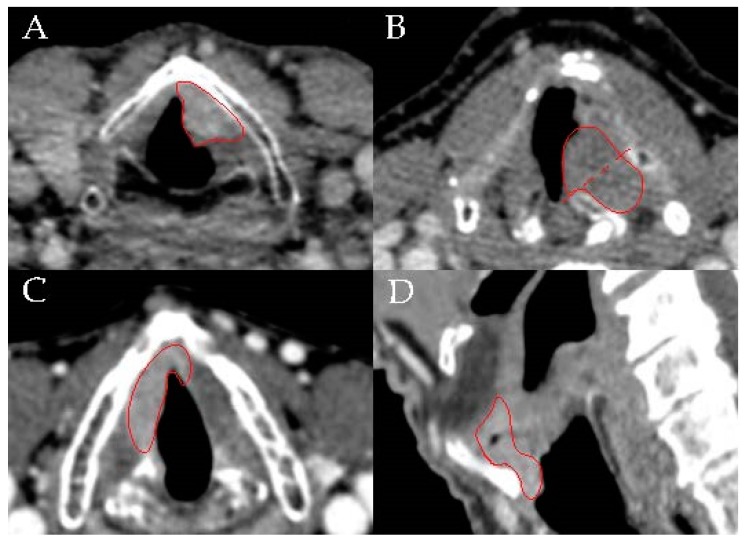
Examples of the four parameters scored in Computed Tomography and Magnetic Resonance Imaging scans.: (**A**) deep infiltration of left glottic in the vocal fold muscle; (**B**) tumor that is located both anterior and posterior of the M-line; (**C**) horizontal involvement of the anterior commissure in the glottic plane; (**D**) vertical involvement of the anterior commissure (supraglottic and infraglottic extension).

We retrospectively reviewed the records of all patients diagnosed with T2N0 glottic squamous cell carcinoma that received primary treatment with radiotherapy at the University Cancer Centre Leiden—the Hague between 2000 and 2012. The cancer center has two locations, one in Leiden and one in the Hague, which use the same irradiation schemes/protocols. Patients were identified through the hospital oncological database, which registers the site and the stage of all patients with oncological tumors. We assessed the medical charts of these patients and collected data on the demographics, tumor characteristics, diagnostics, treatment details, follow-up, and patient outcomes. All available Computed Tomography (CT) or Magnetic Resonance Imaging (MRI) scans were reviewed by the radiologist (B.M.V.) and scored for several variables related to tumor location: 1) superficial versus deep vocal fold muscle infiltration; 2) anterior versus posterior vocal fold muscle involvement defined by the relation of the tumor to the vertical plane tangential to the arytenoid vocal process and perpendicular to the ipsilateral thyroid lamina (henceforth: position relative to the M-line as defined by Succo et al. [10]); 3) horizontal involvement of the anterior commissure in the glottic plane; and 4) vertical involvement of the anterior commissure resulting from supraglottic and subglottic extension (Figure 1). If the tumors were not visible on CT or MRI, they were classified as superficial. In the patients where the original scans were not available for re-evaluation, radiological reports were reviewed to rule out paraglottic or pre-epiglottic space involvement or suspicious neck nodes. In three patients, imaging had not been performed. All these three patients had normal mobility of the vocal folds and N0 neck on ultrasound and were therefore included in the cohort. Acute and chronic radiotherapy toxicities were analyzed according to the Common Terminology Criteria for Adverse Events, version 4.0 (National Cancer Institute, Bethesda, USA). Patients were excluded if they had previous laryngeal cancer or received a primary treatment other than radiotherapy. The study was approved by the Leiden University Medical Center with approval number G17.098/SH/sh on 13th February 2018.

### 2.2. Follow-Up

During radiotherapy, patients underwent a weekly clinical examination. After treatment, all patients were periodically evaluated, according to the protocol, with flexible fiberoptic laryngoscopy. Evaluations were scheduled every two months in the first year and with decreasing frequency until five years posttreatment. When a suspected lesion was detected during follow-up, a biopsy was performed. 

### 2.3. Statistical Analysis 

Data analyses were performed with SPSS version 23.0 (IBM Corp., Armonk, NY, USA). The outcome parameters of the study were the five- and 10-year rates of LC, disease-specific survival (DSS), overall survival (OS), and LP. The median follow was calculated with the reverse Kaplan–Meier method. The Kaplan–Meier five- and 10-year survival curves were used to calculate LC, DSS, OS, and LP. The entry point was the start of radiotherapy. The endpoints were the date of the first local recurrence, for LC; the date of death due to laryngeal cancer, for DSS; the date of death of all causes, for OS; and the date of the laryngectomy, for LP. Th potential prognostic factors were evaluated with univariate analysis and the log-rank test. The following variables were tested in univariate models for LC, DSS, and LP: involvement of the anterior commissure, mobility of the vocal cords, tumor infiltration relative to the vocal fold muscle, tumor position relative to the M-line, horizontal and vertical involvement of the anterior commissure, type of radiotherapy (conventional versus intensity-modulated radiation therapy (IMRT)), elective lymph node irradiation, total dose, fraction size, and overall treatment time. Multivariate analysis using cox proportional hazard analysis was not performed due to insufficient sample size. Bonferroni correction was applied to correct for multiple testing (Bonferroni-corrected value of *p* = 0.05/11 = 0.005).

## 3. Results

### 3.1. Patients and Treatment Characteristics 

Between 2000 and 2012, 94 consecutive patients were treated with radiotherapy for cT2N0 glottic carcinoma. There were 82 male (87.2%) and 12 (12.8%) female patients. Mean age at diagnosis was 64.4 (range 32–84) years. Seventy-nine patients (84%) were treated with conventional radiotherapy, applied as a parallel-opposed bilateral field, generated with a 4–6 MV linear accelerator. Fifteen patients (16%) were treated with IMRT. The total dose ranged from 60.0–70.0 Gy (median 68.0 Gy); the dose per fraction ranged from 1.8 to 2.4 Gy (median 2.0 Gy). In total, 37 patients (39.4%) underwent elective lymph node irradiation, with a total dose range of 44.0 to 58.0 Gy (median 46.0 Gy). The overall treatment time ranged from 35 to 49 days (median 42.0 days). 

### 3.2. Radiological Characteristics

In 50 patients, CT or MRI scans were available. Four scans were excluded because of movement and streaking artifacts. In total, 46 scans were included for the analysis, of which 36 were CT (78.2%), and 10 were MRI (21.7%) scans. Of the 46 included scans, 31 were of good quality and 15 of moderate quality. The radiological characteristics are listed in Table 1. 

### 3.3. Follow-Up 

Three patients (3.2%) were lost to follow-up at two months, five months, and six months, respectively. These three patients were clinically free of disease at the time of their last examination. The median follow-up was 106.0 months (95%CI: 80.3–131.7) (range 2–175 months). 

### 3.4. Survival and Local Control

**Figure 2:**
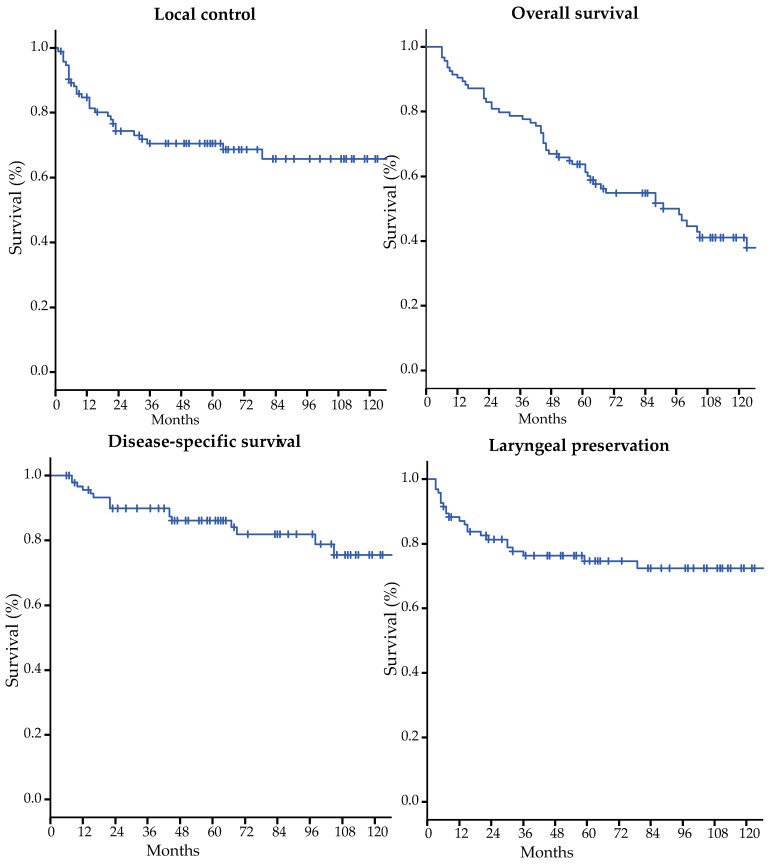
Oncological outcomes.

During follow-up, 28 patients (29.8%) developed local recurrence, after a median of 11.5 months (range 1–79 months). Of these, recurrence was detected within 24 months of treatment in 82.1%. The five- and 10-year LC rates were 70.5% and 65.8%, respectively (Table 2 and Figure 2). Salvage therapy consisted of TLM in three patients (10.7%); re-irradiation in two patients (7.1%); total laryngectomy with or without neck dissection, including radiation of the neck in 18 patients (67.9%), and partial laryngectomy in two patients (7.1%). Additionally, two patients (7.1%) required a total laryngectomy, but refused this, and therefore, received the best supportive care. None of our patients had neck failure without having a local recurrence. In six of the 28 patients (21%) that developed a local recurrence, positive neck nodes were identified. Two of the six patients had undergone elective neck irradiation during primary treatment, and four patients had not. Ten patients (35.7%) developed a second recurrence. Of these, two patients were treated by re-irradiation; two patients underwent a total laryngectomy with neck dissection; one patient underwent a total pharyngectomy and a gastric tube reconstruction with a pectoralis major muscle flap; two patients underwent surgical resection, and one with and one without radiotherapy, due to stomal recurrence. Three patients did not have curative treatment, of which one patient underwent palliative chemotherapy, and two patients received the best supportive care. In total, 23 patients (24.5%) underwent a total laryngectomy due to recurrent disease. The five- and 10-year LP rates were 74.4% and 72.4%, respectively (Table 2 and Figure 2). During follow-up, 16 patients died due to laryngeal cancer (17.0%), and 38 patients (40.4%) died of unrelated causes. The five- and 10-year DSS rates were 86.0 and 75.6%, and the five- and 10-year OS rates were 63.7 and 41.0% (Table 2 and Figure 2). In the univariate analysis, one radiological variable (vertical involvement of the anterior commissure) showed a significant association with LC and one radiological variable (horizontal involvement) showed a trend towards lower LC (Table 1). All four patients with sub- and supraglottic extension in the anterior commissure developed recurrent disease, (five-year LC 0%). Horizontal involvement of the anterior commissure also showed a trend towards lower LP (*p* = 0.009), with a five-year LP of 59.2% in patients with horizontal anterior commissure involvement versus 90.1% in those without. Deep infiltration of the vocal muscle showed a trend towards lower DSS (*p* = 0.037) with a five-year DSS of 57.5% in patients with deep infiltration versus 79.2% in those with only superficial involvement and a trend towards higher LC in patients with superficial tumor infiltration (*p* = 0.077) (Table 1). No other clinical or treatment-related variable showed any significant impact on the oncological outcomes.

### 3.5. Toxicity 

No patient died due to toxicity from radiotherapy. Overall, thirteen patients (13.8%) reported grade 3 or grade 4 adverse events. Acute adverse events were reported in 10 patients: in one patient, radiotherapy was interrupted due to laryngeal edema, which required a tracheostomy (grade 4) and nine patients required a nasogastric feeding tube during radiation therapy for grade 3 dysphagia. Five patients (5.3%) had late complications (grades 2 and 4): two patients developed laryngeal necrosis for which they underwent a tracheostomy, and three patients required treatment for hypothyroidism.

## 4. Discussion 

The objective of this retrospective study was to evaluate the five- and 10-year survival outcomes of patients primarily treated with radiotherapy for T2N0 glottic carcinoma and to identify prognostic factors associated with radiotherapy failure in these patients. Treatment with radiotherapy alone resulted in good outcomes rates at five- and 10-years for LC (70.5% and 65.8%), OS (63.7% and 41.0%), DSS (86.0% and 75.6%), and LP (74.7% and 72.4%). Despite the small numbers of available scans (*n* = 46), vertical anterior commissure involvement on imaging showed a significant impact on LC. No other patient, tumor, treatment, or radiological-related variable in our analysis had any significant impact on oncological outcomes.

Primary radiotherapy is a widely accepted treatment option for early glottic cancer. It is the advocated treatment in the Netherlands for extended T1a tumors and T2 tumors. In the literature, the five-year LC rate for T2 tumors ranges from 48 to 97.6%, with a weighted average of 75.81% [5]. These wide ranges probably reflect the heterogeneity of tumor extension and location found within the T2 stage [11]. This study showed five-year LC rates comparable to those reported in the literature. Our five-year OS and DSS rates were also comparable to those in the literature (ranges 53–91% and 69–100%, respectively) [12,13,14,15,16,17,18,19,20,21,22,23,24,25,26,27,28]. 

Only a few studies have reported 10-year oncological outcomes for T2 glottic carcinoma treated with radiotherapy. Khan et al. reported their 20-year experience of definitive radiotherapy for early glottic cancer. They divided T2 tumors into T2a (with mobile vocal folds) and T2b (without mobile vocal folds) types and showed LC rates of 87% and 56%, respectively. Their 10-year OS for T1–T2 tumors was 50% [29]. Chera et al. reported 10-year OS rates of 51% and 49% for T2a and T2b tumors, respectively [17]. Le et al. reported a 10-year OS of 63% for T2 tumors [30]. Frata et al. reported 10-year LC, OS, and DSS of 70%, 37%, and 85%, respectively [31]. These 10-year oncological outcomes are comparable with those found in this study. 

Several retrospective studies have investigated prognostic factors for radiation failure in early glottic carcinoma. However, to date, little evidence has been published from randomized controlled trials or large prospective studies. Recently, a systematic review with a meta-analysis investigated 56 potential risk factors for radiation failure in early glottic carcinoma (T1–T2) [32]. Its results indicate that male gender, low hemoglobin level, anterior commissure involvement, tumor stage (T2 tumor), tumor size/volume, and poor differentiation/dedifferentiation could increase the probability of radiation failure. Several studies have focused on the involvement of the anterior commissure as a potential risk factor for radiation failure. Although it is widely acknowledged that the involvement of the anterior commissure can have a negative impact on the outcome, the extent of the impact remains a topic of discussion, with the results reported in literature being inconsistent. Some studies show an association between the anterior commissure involvement and LC [33,34,35,36,37], whereas others do not [12,22,27,29,30,31,38,39]. In our study, we found that the clinical, binary variable on anterior commissure involvement (yes/no) had no significant impact on oncological outcomes, whereas the vertical involvement of the anterior commissure on imaging showed a significant impact on LC. The varying results found in the literature can be explained by the variations in clinical definition of the anterior commissure area, variation in the detail of the clinical, endoscopic, and radiological evaluation of the lesion in the preoperative setting, the distinctive features, and limitation of each therapeutic modality, the biological behaviors of the tumor, and variations in the rigor of the follow-up policy [40]. These factors, combined with the complicated anatomy of the anterior commissure, the involvement of this subsite may very well be too complex to be included as a binary variable (yes/no) [40]. Therefore, the role of the anterior commissure in the risk of primary radiotherapy failure in patients with T2 glottic carcinoma remains unclear, and further studies are needed to elucidate the impact of this sublocalization on the outcome. 

The inconsistency in the literature regarding the impact of anterior commissure involvement is also found in surgical series (TLM). Notably, T2 tumors with vertical anterior commissure extension (supracommissural and subcommissural) have a significantly lower LC and LP rate with TLM than other T2 subtypes [11,41,42]. Also, in T3 tumors, the involvement of the posterior part of the muscle, behind the so-called M-line, has been associated with a significantly lower LC, DSS, and OS rate in patients treated with open partial horizontal laryngectomies [10]. The higher risk of oncological failure in these areas is thought to be related to their proximity to some of the membranes and visceral spaces of the larynx such as the pre- and paraglottic space and the cricothyroid ligament. 

To the best of our knowledge, this is the first study to investigate these surgical risk factors in a radiotherapy cohort. In accordance with the aforementioned surgical studies, we found a significant impact of vertical involvement of the anterior commissure on LC and a trend towards lower LC and LP in the horizontal involvement of the anterior commissure as classified on imaging. Deep infiltration in the vocal fold muscle also showed a trend towards lower LC and DSS. Although these trends were not significant in this study, we view our findings as an indication that these parameters warrant further study. The number of patients in the different radiological categories was limited, especially in the case of the vertical anterior commissure involvement. In this subgroup, none of the patients with supraglottic extension in the anterior commissure developed a local recurrence versus 25% of patients with subglottic extension and 100% of patients with sub- and supraglottic extension. Currently, we have no evident explanation for this. However, it might be that the involvement of these sites carries increasing levels of risk. This theory has to be investigated in the future, prospective studies to extend the understanding of these variables. Therefore, these results have to be interpreted with caution. 

To further improve LC rates in high-risk patients in T2 glottic carcinoma, chemoradiotherapy (CRT) might be considered. Several studies have investigated this treatment modality in T2 laryngeal cancer. They show that CRT is feasible, well-tolerated, and effective. Furthermore, they show promising LC rates between 91.5% and 100% [43,44,45,46,47]. However, the number of patients treated with CRT was low, and some studies also included T1 tumors. Three studies compared RT with CRT [45,48,49]. One of these studies concluded that CRT was not found to be effective for 21 patients with T2 glottic carcinoma [49]. However, the study of Akimoto et al. reported a significant difference in the five-year disease-free survival between RT alone and CRT (68% and 89%, respectively) [48]. The study by Bhateja et al. compared the outcome of patients with T2bN0 tumors treated with RT to that of patients with T2b-T3N0/N+ treated with CRT. They found that the T2bN0 tumors treated with RT alone showed a trend towards lower LC than the T2b-T3N0/N+ patients treated with CRT, even though the latter group includes higher stage tumors [45]. Thus, data suggest that applying concurrent chemoradiotherapy may be a reasonable strategy to improve LC in patients with high risk T2 glottic carcinoma, although risks and benefit have to be considered [45]. Therefore, a prospective randomized controlled trial is needed to evaluate risks and the improvement of LC in CRT in T2 glottic carcinoma. 

We interpret our results as an important indication that tumor extension in the anterior commissure is a risk factor for reduced oncological control both in surgical and radiotherapy patients and that patients with tumors with these high-risk growth patterns require close surveillance, independently of treatment modality. Due to the retrospective nature of this data, the impact of subcategorization of T2 tumors according to these factors should be further studied in a prospective manner. 

In the aforementioned surgical studies, the detailed classification of the type of anterior commissure involvement was obtained using a combination of clinical, radiological, and surgical information, whereas we based our classification on imaging. This was due to the retrospective nature of this study with which anterior commissure involvement could only be specified as a binary variable (yes/no) and could not be further determined, whereas the imaging could be reevaluated specifically for this study. As stated earlier, the binary involvement of the anterior commissure based on our clinical information did not have a significant impact on LC. Additionally, we did not find a significant impact of the mobility of the vocal fold, whereas deep involvement demonstrated a trend towards increased LC. This suggests that a more specific classification of tumors in this area than is currently offered by the TNM classification system, may be necessary [41,42] and that incorporating imaging improves evaluation of tumor extent.

## 5. Limitations

This study has some limitations. First, the sample size was small, especially the numbers of scans that were available (*n* = 46). This means that our findings are based on a small number of observations. Larger studies are needed to confirm these preliminary findings. Also, the retrospective nature of the study meant that we would not obtain all the scans for reevaluation and had to rely on reports of the imaging for the confirmation of the tumor stage (T2N0). In addition, due to the retrospective design, it was not possible to include all the risk factors mentioned in the literature, due to a lack of available data and the low numbers of events. 

## 6. Conclusions

This study shows good oncological outcomes for patients with T2 glottic carcinoma treated with radiotherapy at our center. In accordance with previously described subtypes of T2 glottic carcinoma and known surgical risk factors, we identified vertical involvement of the anterior commissure on imaging as a prognostic factor for radiation failure. Prospective studies are warranted to extend our understanding of tumor extension variables on imaging in all treatment modalities for glottic cancer. 

## Figures and Tables

**Table 1:** Univariate analysis for impact on local control.

Characteristics	No. of Patients (%)	5-Year Local Control (%)	*p*-Value
**Clinical characteristics (*n* = 94)**			
Involvement AC			0.597
Yes	61 (64.9)	68.1	
No	33 (35.1)	74.8	
Mobility			0.438
Normal	76 (80.9)	69.1	
Impaired	18 (19.1)	77.4	
**Radiological characteristics (*n* = 46)**			
Tumor infiltration in VM			0.077
Superficial	16 (34.8)	92.9	
Deep	30 (65.2)	68	
Position relative to M-line			0.875
Anterior	37 (82.2)	76.6	
Posterior	1 (2.2)	100	
Both	7 (15.6)	71.4	
Horizontal involvement AC			
Yes	13 (28.3)	59.2	0.047
No	33 (71.7)	83	
Vertical involvement AC			<0.0001
No	32 (71.1)	81.8	
Supraglottic	5 (11.1)	100	
Subglottic	4 (8.9)	75	
Both	4 (8.9)	0	
**Treatment characteristics (*n* = 94)**			
Type of radiotherapy			0.277
Normal	79 (84.0)	71.6	
IMRT	15 (16.0)	65.2	
Elective neck irradiation			0.827
Yes	37 (39.4)	68.6	
No	57 (60.6)	72	
Total dose			0.965
≤68 Gy	49 (52.1)	71.8	
>68 Gy	45 (47.9)	69.1	
Fraction size			0.77
≤2.0 Gy	84 (89.4)	71	
>2.0 Gy	10 (10.6)	67.5	
Overall treatment time			0.331
≤42 days	73 (77.7)	68.2	
>42 days	21 (22.3)	79	

Abbreviations: AC= anterior commissure, Gy = gray, IMRT= intensity-modulated radiation therapy, VM = vocal fold muscle.

**Table 2:** Five- and ten-year oncological outcomes.

Outcomes	Five-Year Survival (%)	10-Year Survival (%)
Local control	70.5	65.8
Overall survival	63.7	41.0
Disease-specific survival	86.0	75.6
Laryngeal preservation	74.7	72.4

## References

[1] Nederlandse Kankerregistratie (NKR), IKNL. https://www.cijfersoverkanker.nl/selecties/Dataset_1/img5b756230c37f1.

[2] Sjögren E.V. (2006). Epidemiology of head and neck squamous cell carcinoma. Prognosis in Head and Neck Cancer.

[3] Nederlandse Werkgroep Hoofd-Halstumoren NWHHT (2010). Larynxcarcinoom Inhoudsopgave.

[4] Warner L., Chudasama J., Kelly C.G., Loughran S., McKenzie K., Wight R., Dey P., Arnold D., Wight R., MacKenzie K. (2014). Radiotherapy versus open surgery versus endolaryngeal surgery (with or without laser) for early laryngeal squamous cell cancer. Cochrane Database Syst. Rev..

[5] Warner L., Lee K., Homer J.J. (2017). Transoral laser microsurgery versus radiotherapy for T2 glottic squamous cell carcinoma: A systematic review of local control outcomes. Clin. Otolaryngol..

[6] Mo H.L., Li J., Yang X., Zhang F., Xiong J.W., Yang Z.L., Tan J., Li B. (2017). Transoral laser microsurgery versus radiotherapy for T1 glottic carcinoma: A systematic review and meta-analysis. Lasers Med. Sci..

[7] Abdurehim Y., Hua Z., Yasin Y., Xukurhan A., Imam I., Yuqin F. (2012). Transoral laser surgery versus radiotherapy: Systematic review and meta-analysis for treatment options of T1a glottic cancer. Head Neck.

[8] Schrijvers M.L., van Riel E.L., Langendijk J.A., Dikkers F.G., Schuuring E., van der Wal J.E., van der Laan B.F. (2009). Higher laryngeal preservation rate after CO2 laser surgery compared with radiotherapy in T1a glottic laryngeal carcinoma. Head Neck.

[9] Hendriksma M., Heijnen B.J., Sjögren E.V. (2018). Oncologic and functional outcomes of patients treated with transoral CO2 laser microsurgery or radiotherapy for T2 glottic carcinoma: A systematic review of the literature. Curr. Opin. Otolaryngol. Head Neck Surg..

[10] Succo G., Crosetti E., Bertolin A., Piazza C., Molteni G., Cirillo S., Petracchini M., Tascone M., Sprio A.E., Berta G.N. (2018). Treatment for T3 to T4a laryngeal cancer by open partial horizontal laryngectomies: Prognostic impact of different pathologic tumor subcategories. Head Neck.

[11] Peretti G., Piazza C., Mensi M.C., Magnoni L., Bolzoni A. (2005). Endoscopic treatment of cT2 glottic carcinoma: Prognostic impact of different pT subcategories. Ann. Otol. Rhinol. Laryngol..

[12] Chen M.F., Chang J.T., Tsang N.M., Liao C.T., Chen W.C. (2003). Radiotherapy of early-stage glottic cancer: Analysis of factors affecting prognosis. Ann. Otol. Rhinol. Laryngol..

[13] Dagan R., Morris C.G., Bennett J.A., Mancuso A.A., Amdur R.J., Hinerman R.W., Mendenhall W.M. (2007). Prognostic significance of paraglottic space invasion in T2N0 glottic carcinoma. Am. J. Clin. Oncol. Cancer Clin. Trials.

[14] Sakata K., Oouchi A., Nagakura H., Akiba H., Tamakawa M., Koito K., Hareyama M., Asakura K., Satoh M., Ohtani S. (2000). Accelerated radiotherapy for T1, 2 glottic carcinoma: Analysis of results with KI-67 index. Int. J. Radiat. Oncol. Biol. Phys..

[15] Smee R., Bridger G.P., Williams J., Fisher R. (2000). Early glottic carcinoma: Results of treatment by radiotherapy. Australas. Radiol..

[16] Garden A.S., Forster K., Wong P.F., Morrison W.H., Schechter N.R., Ang K.K. (2003). Results of radiotherapy for T2N0 glottic carcinoma: Does the “2” stand for twice-daily treatment?. Int. J. Radiat. Oncol. Biol. Phys..

[17] Chera B.S., Amdur R.J., Morris C.G., Kirwan J.M., Mendenhall W.M. (2010). T1N0 to T2N0 squamous cell carcinoma of the glottic larynx treated with definitive radiotherapy. Int. J. Radiat. Oncol. Biol. Phys..

[18] Jones D.A., Mendenhall C.M., Kirwan J., Morris C.G., Donnan A., Holwerda S., Kraus S.T., Mann C.J., Grant J.R., Donnan B. (2010). Radiation therapy for management of t1-t2 glottic cancer at a private practice. Am. J. Clin. Oncol..

[19] Tong C.C., Au K.H., Ngan R.K., Cheung F.Y., Chow S.M., Fu Y.T., Au J.S., Law S.C. (2012). Definitive radiotherapy for early stage glottic cancer by 6 MV photons. Head Neck Oncol..

[20] Hoebers F., Rios E., Troost E., van den Ende P., Kross K., Lacko M., Lalisang R., Kremer B., De J.J. (2013). Definitive radiation therapy for treatment of laryngeal carcinoma: Impact of local relapse on outcome and implications for treatment strategies. Strahlenther. Onkol..

[21] Furusaka T., Matsuda H., Saito T., Katsura Y., Ikeda M. (2012). Long-term follow-up and salvage surgery in patients with T2N0M0 squamous cell carcinoma of the glottic larynx who received concurrent chemoradiation therapy with carboplatin (CBDCA) AUC 1.5 vs AUC 2.0. Acta Otolaryngol..

[22] Gorphe P., Blanchard P., Breuskin I., Temam S., Tao Y., Janot F. (2015). Vocal fold mobility as the main prognostic factor of treatment outcomes and survival in stage II squamous cell carcinomas of the glottic larynx. J. Laryngol. Otol..

[23] Harada A., Sasaki R., Miyawaki D., Yoshida K., Nishimura H., Ejima Y., Kitajima K., Saito M., Otsuki N., Nibu K. (2015). Treatment outcomes of the patients with early glottic cancer treated with initial radiotherapy and salvaged by conservative surgery. Jpn. J. Clin. Oncol..

[24] Motegi A., Kawashima M., Arahira S., Zenda S., Toshima M., Onozawa M., Hayashi R., Akimoto T. (2015). Accelerated radiotherapy for T1 to T2 glottic cancer. Head Neck.

[25] Murakami R., Nishimura R., Baba Y., Furusawa M., Ogata N., Yumoto E., Yamashita Y. (2005). Prognostic factors of glottic carcinomas treated with radiation therapy: Value of the adjacent sign on radiological examinations in the sixth edition of the UICC TNM staging system. Int. J. Radiat. Oncol. Biol. Phys..

[26] Shor S., Krawitz H., Macann A., West T., Morton R.P., Mcivor N.P., Chaplin J., Simcock P., Gathercole J., Dorman B. (2006). T1N0/T2N0glottic carcinoma: A comparison of two fractionation schedules. Australas. Radiol..

[27] Stoeckli S.J., Schnieper I., Huguenin P., Schmid S. (2003). Early glottic carcinoma: Treatment according patient’s preference?. Head Neck.

[28] Raitiola H., Wigren T., Pukander J. (2000). Radiotherapy outcome and prognostic factors in early glottic carcinoma. Auris Nasus Larynx.

[29] Khan M.K., Koyfman S.A., Hunter G.K., Reddy C.A., Saxton J.P. (2012). Definitive radiotherapy for early (T1-T2) glottic squamous cell carcinoma: A 20 year Cleveland Clinic experience. Radiat. Oncol..

[30] Le Q.T.X., Fu K.K., Kroll S., Ryu J.K., Quivey J.M., Meyler T.S., Krieg R.M., Phillips T.L. (1997). Influence of fraction size, total dose, and overall time on local control of T1-T2 glottic carcinoma. Int. J. Radiat. Oncol. Biol. Phys..

[31] Frata P., Cellai E., Magrini S.M., Bonetti B., Vitali E., Tonoli S., Buglione M., Paiar F., Barca R., Fondelli S. (2005). Radical radiotherapy for early glottic cancer: Results in a series of 1087 patients from two Italian radiation oncology centers. II. The case of T2N0 disease. Int. J. Radiat. Oncol. Biol. Phys..

[32] Eskiizmir G., Baskin Y., Yalcin F., Ellidokuz H., Ferris R.L. (2016). Risk factors for radiation failure in early-stage glottic carcinoma: A systematic review and meta-analysis. Oral Oncol..

[33] Bron L.P., Soldati D., Zouhair A., Ozsahin M., Brossard E., Monnier P., Pasche P. (2001). Treatment of early stage squamous-cell carcinoma of the glottic larynx: Endoscopic surgery or cricohyoidoepiglottopexy versus radiotherapy. Head Neck.

[34] Zouhair A., Azria D., Coucke P., Matzinger O., Bron L., Moeckli R., Do H.P., Mirimanoff R.O., Ozsahin M. (2004). Decreased local control following radiation therapy alone in early-stage glottic carcinoma with anterior commissure extension. Strahlenther. Onkol..

[35] Nur D.A., Oguz C., Kemal E.T., Ferhat E., Sulen S., Emel A., Munir K., Ann C.S., Mehmet S. (2005). Prognostic factors in early glottic carcinoma implications for treatment. Tumori.

[36] Marshak G., Brenner B., Shvero J., Shapira J., Ophir D., Hochman I., Marshak G., Sulkes A., Rakowsky E. (1999). Prognostic factors for local control of early glottic cancer: The Rabin Medical Center retrospective study on 207 patients. Int. J. Radiat. Oncol. Biol. Phys..

[37] Burke L.S., Greven K.M., McGuirt W.T., Case D., Hoen H.M., Raben M. (1997). Definitive radiotherapy for early glottic carcinoma: Prognostic factors and implications for treatment. Int. J. Radiat. Oncol. Biol. Phys..

[38] Al-Mamgani A., van Rooij P.H., Woutersen D.P., Mehilal R., Tans L., Monserez D., Baatenburg de Jong R.J. (2013). Radiotherapy for T1-2N0 glottic cancer: A multivariate analysis of predictive factors for the long-term outcome in 1050 patients and a prospective assessment of quality of life and voice handicap index in a subset of 233 patients. Clin. Otolaryngol..

[39] Bignardi M., Antognoni P., Sanguineti G., Magli A., Molteni M., Merlotti A., Richetti A., Tordiglione M., Conte L., Magno L. (2004). Hyperfractionated radiotherapy for T2N0 glottic carcinoma: A retrospective analysis at 10 years follow-up in a series of 60 consecutive patients. Tumori.

[40] Hendriksma M., Sjogren E.V. (2019). Involvement of the Anterior Commissure in Early Glottic Cancer (Tis-T2): A Review of the Literature. Cancers.

[41] Piazza C., Filauro M., Paderno A., Marchi F., Perotti P., Morello R., Taboni S., Parrinello G., Incandela F., Iandelli A. (2018). Three-Dimensional Map of Isoprognostic Zones in Glottic Cancer Treated by Transoral Laser Microsurgery as a Unimodal Treatment Strategy. Front. Oncol..

[42] Carta F., Bandino F., Olla A.M., Chuchueva N., Gerosa C., Puxeddu R. (2018). Prognostic value of age, subglottic, and anterior commissure involvement for early glottic carcinoma treated with CO2 laser transoral microsurgery: A retrospective, single-center cohort study of 261 patients. Eur. Arch. Otorhinolaryngol..

[43] Nonoshita T., Shioyama Y., Nakamura K., Nakashima T., Ohga S., Yoshitake T., Ohnishi K., Terashima K., Asai K., Honda H. (2010). Concurrent chemoradiotherapy with S-1 for T2N0 Glottic squamous cell carcinoma. J. Radiat. Res..

[44] Kimura K., Itoh Y., Okada T., Nakahara R., Kawamura M., Kubota S., Itoh J., Hiramatsu M., Fujimoto Y., Shibata T. (2015). Critical evaluation of a prospective study of concurrent chemoradiotherapy with S-1 for early glottic carcinoma. Anticancer Res..

[45] Bhateja P., Ward M.C., Hunter G.H., Greskovich J.F., Reddy C.A., Nwizu T.I., Lamarre E., Burkey B.B., Adelstein D.J., Koyfman S.A. (2016). Impaired vocal cord mobility in T2N0 glottic carcinoma: Suboptimal local control with Radiation alone. Head Neck.

[46] Kimura K., Itoh Y., Okada T., Kubota S., Kawamura M., Nakahara R., Oie Y., Kozai Y., Takase Y., Tsuzuki H. (2017). Optimized treatment strategy of radiotherapy for early glottic squamous cell carcinomas: An initial analysis. Nagoya J. Med. Sci..

[47] Saitoh J.-I., Shirai K., Imaeda M., Musha A., Abe T., Shino M., Takayasu Y., Takahashi K., Chikamatsu K., Nakano T. (2017). Concurrent chemoradiotherapy with conventional fractionated radiotherapy and low-dose daily cisplatin plus weekly docetaxel for T2N0 glottic cancer. Radiat. Oncol..

[48] Akimoto T., Nonaka T., Kitamoto Y., Ishikawa H., Ninomiya H., Chikamatsu K., Furuya N., Hayakawa K., Mitsuhashi N., Nakano T. (2006). Radiation therapy for T2N0 laryngeal cancer: A retrospective analysis for the impact of concurrent chemotherapy on local control. Int. J. Radiat. Oncol. Biol. Phys..

[49] Kitani Y., Kubota A., Furukawa M., Sato K. (2016). Prognostic factors for local control in patients receiving radiation therapy for early glottic cancer: Anterior commissure involvement and effect of chemoradiotherapy. Eur. Arch. Otorhinolaryngol..

